# “You Are Left Holding Their Despair”: A Reflexive Thematic Analysis of Secondary Trauma Among School Staff

**DOI:** 10.1007/s40653-026-00972-6

**Published:** 2026-09-09

**Authors:** Paul Gordon, Martin Dempster, John McMullen, Donncha Hanna

**Affiliations:** Queen’s University Belfast, Belfast, United Kingdom

**Keywords:** Secondary Traumatic Stress, Educator Well-being, Trauma-Informed Schools, Vicarious Resilience, Reflexive Thematic Analysis, Childhood Trauma Exposure

## Abstract

**Background:**

School personnel are frequently exposed to student trauma, placing them at risk for secondary traumatic stress (STS) and potentially compromising the school’s capacity to effectively support traumatized youth. However, the conceptualization of STS within education remains ambiguous, and the experiences of staff, particularly in Ireland and the U.K., are underexplored. This study aimed to explore the lived experiences of secondary trauma among school personnel in Ireland and the U.K. to understand its effects and identify sources of perceived support.

**Method:**

Semi-structured online interviews were conducted with 11 school personnel, purposively sampled from primary and secondary schools in the U.K. and Ireland. Data were analyzed using reflexive thematic analysis, within a constructivist paradigm.

**Findings:**

Four main themes were constructed: (1) A System Under Strain, detailing institutional failures and performative support; (2) The Intrusive Weight of Trauma, describing the emotional and personal toll, including STS symptoms like intrusion and mood changes; (3) The Nurturer and The Fixer, exploring the negotiation of professional identity and personal responsibility toward trauma-impacted students; and (4) Navigating the Burden, a patchwork of informal coping mechanisms in the absence of formal support.

**Conclusion:**

STS is a significant occupational risk for educators, potentially exacerbated by systemic failures. The findings suggest that school staff experience some symptoms consistent with STS. However, not all criteria indicative of clinical levels are represented, and the concept of ''mirrored trauma'' is proposed to describe how staff’s personal histories and identities may shape the unique manifestation of STS in this population. Staff call for a shift from a focus on individual resilience to creating system-wide, trauma-informed support structures. This systemic support is argued to be essential for both staff well-being and child trauma recovery. Potential implications for school policy are discussed.

**Supplementary Information:**

The online version contains supplementary material available at https://doi.org/10.1007/s40653-026-00972-6.

## Introduction

Trauma and adverse experiences are common among school-aged children, putting them at risk for behavior and learning problems at school (Avery et al., 2021; Beasley & Norris, 2021; Essary et al., 2020). Additionally, the high prevalence of trauma among students means that teachers and other school staff often deal with its effects through their interactions with students, which can include hearing about or knowing details of students’ traumatic events (Christian-Brandt et al., 2020). To address the prevalence of student trauma, there have been calls to implement trauma-informed practices in schools that recognize the impact of trauma and develop strategies to prevent its spread among students (Aragón et al., 2024; Dorado et al., 2016; Heck et al., 2023; Yohannan & Carlson, 2019). One framework for operationalizing such practices is the Substance Abuse and Mental Health Services Administration’s (SAMHSA, 2014) model of trauma-informed care, which identifies six core principles (safety, trustworthiness and transparency, peer support, collaboration and mutuality, empowerment, voice, and choice, and cultural, historical, and gender issues) as foundational to organizational environments in which those affected by trauma may thrive. However, this presents a challenge, as teachers in countries like the United States, United Kingdom, and Australia often report poor well-being (Doan et al., 2024; Education Support, 2025) and can feel unprepared to support trauma-exposed students (Brunzell et al., 2021). These reports indicate that many school staff are not being provided with the necessary resources and support to operate effectively, while maintaining their health and well-being. Moreover, research from these same countries suggests that interactions with trauma-affected youth who may display behavioral issues can increase teachers'' occupational stress, reduce their well-being, and contribute to burnout, often leading them to leave their positions prematurely (Essary et al., 2020; Fazel et al., 2014; Oberg et al., 2023).

Evidence suggests that an essential factor contributing to school personnel’s poor well-being and attrition stems from hearing about their students’ trauma experiences (Borntrager et al., 2012; Caringi et al., 2015; Christian-Brandt et al., 2020; Essary et al., 2020). The current study examines a key health effect that school staff can develop from the emotional burden associated with hearing or knowing about their students’ trauma, called Secondary Traumatic Stress (STS). STS is conceptualized as a group of psychological symptoms that resemble those of post-traumatic stress disorder (PTSD), though it derives from indirect exposure to the trauma of others whom one seeks to help (Figley, 1995). Symptoms of STS can include intrusion, changes in mood and cognition, changes in reactivity and arousal, and avoidance (Fleckman et al., 2022). STS symptoms may manifest in school staff as strong emotional reactions and irritability towards students, sleep disruption, and worries about student safety (Hydon et al., 2015), as well as changes in one’s view of self or others (Figley, 1995). These symptoms can seriously impact teachers’ engagement with students and their job performance (Lawson et al., 2019). Therefore, addressing STS among school personnel is crucial to improving staff health and well-being and students'' educational outcomes.

Despite increasing research into the effects of secondary trauma, there is no consistently adopted definition of STS (Sprang et al., 2019). Instead, the concept is described through several overlapping terms rooted in different theoretical models. A foundational theory by Figley (1995) defines STS as a natural emotional and behavioral response to knowing about a significant other’s traumatic event, with symptoms that can be “nearly identical to PTSD” (p.8). According to this view, STS and Compassion Fatigue (CF) are interchangeable (Ludick & Figley, 2017). This understanding of STS symptoms, as comparable to PTSD, aligns with the updated Diagnostic and Statistical Manual of Mental Disorders (DSM-5) criteria, which recognizes that secondary exposure to trauma can, in some cases, meet the criteria for PTSD diagnosis (American Psychiatric Association, 2013). The DSM-5 update to PTSD criteria has also prompted changes in how STS symptoms are assessed in some measures. Bride’s (2013) Secondary Traumatic Stress Scale (STSS) DSM-5 revision removed item #5 (‘I felt discouraged about the future’) from scoring calculations and moved several items from the ‘Avoidance’ subscale to the ‘Negative Cognition and Mood’ subscale. This change impacts how STS is constructed and assessed by models advancing the construct as symptoms comparable to PTSD. Specifically, ‘Avoidance’ now strictly reflects a more active orientation of wanting to circumvent “people, places, or things” that remind the individual of their work with traumatized clients, rather than the previous inclusion of emotional numbness (Bride, 2013). Other theories and models have sought to refine Figley’s (1995) definition. For example, Stamm’s (2010) Professional Quality of Life (ProQOL) model describes STS as a fear-based reaction to others’ trauma, and distinguishes it from burnout, which is characterized by job-related exhaustion. Alternatively, McCann and Pearlman (1990) conceptualized the effects of indirect trauma as Vicarious Traumatization (VT), focusing on the long-term changes to a professional''s cognitive schemas and worldview. This conflation of terms among STS, CF, and VT, and the ongoing debate about whether STS is a distinct diagnosis from PTSD (Horesh, 2016), has created a muddled conceptual landscape, making it difficult to accurately understand how the phenomenon manifests, particularly within school settings (Ormiston et al., 2022).

Compounding this conceptual ambiguity is emerging evidence that educators may experience STS and related conditions in unique ways compared to other helping professions (Fleckman et al., 2022). Furthermore, research suggests that compassion fatigue (CF), as conceptualized by Figley (1995) as equivalent to STS, represents a “special form” of burnout among teachers, with effects that can vary by role and experience (Yu et al., 2022, p.1). This tangling of concepts indicates that perhaps the current models and measures, which were developed for STS in other fields, such as nursing and counseling, may not adequately capture the unique experiences of secondary trauma in school personnel. Given the ambiguity in terminology and the potentially unique context of education, there is a clear need to understand secondary trauma from the perspective of school staff themselves. Such an investigation requires rich, experiential data that qualitative methods are uniquely positioned to offer.

## Sociocultural and Systemic Context

Trauma, and indeed the sequelae of secondary trauma, do not typically occur in a vacuum. Rather, these psychological and behavioral states are experienced, appraised, and processed by individuals within cultural and social contexts (Muldoon et al., 2019). School personnel navigate their roles within specific policy environments and organizational cultures that shape what support structures exist, what is normalized, and what might be considered traumatic.

The current study draws on the experiences of personnel from three distinct jurisdictions: the Republic of Ireland (RoI), Northern Ireland (NI), and England, each with its own educational governance structures, accountability cultures, and historical legacies (Kreijkes & Johnson, 2023; Roulston et al., 2023). The historical relationship between England and Ireland has shaped the systemic trajectories of their respective educational environments (Roulston et al., 2023). Meanwhile, Northern Ireland carries a unique contextual weight of “the Troubles,” a decades-long conflict in the 1960s to 1990s that resulted in 3,720 fatalities and an estimated 47,540 casualties in a population of 1.7 million (Muldoon, 2024). This widespread impact on families left trauma that still permeates school environments in this region (Bates & McCully, 2021).

These jurisdictional differences are real and worth acknowledging. However, they may matter less to the psychological experience of STS, given their historical connections, shared structural issues, and religious influence (Roulston et al., 2023; Skerritt, 2019). Other connecting threads, including teacher professional identity, perceived organizational support, and the relational nature of student-facing work, may be as salient as, or more salient than, jurisdictional factors in shaping the STS experience among school personnel. While post-traumatic symptoms and precipitating events may vary by culture, cross-cultural evidence suggests that extreme traumatic experiences, whatever they may be, can trigger psychological distress (Muldoon et al., 2019). Consequently, this study does not aim to compare jurisdictions; it seeks to understand STS as a psychological experience within a participant group that spans all three.

### The Current Study

Built on the previously discussed conceptualizations by Figley (1995) and Stamm (2010), this study defines STS as the distressing or unwanted psychological or behavioral effects arising from indirect exposure to others’ traumatic experiences, which potentially mirror PTSD symptoms. The current study employs a reflexive thematic analysis (RTA) to explore the experiences of school personnel who are indirectly exposed to students’ trauma. Additionally, it examines the effects of indirect trauma exposure and the potential sources of perceived support. The study is grounded on a constructivist paradigm within an interpretivist framework, meaning the researchers see reality as a set of social constructs (relativist ontology) and understand that knowledge is co-created through interactions between researchers and participants (subjectivist epistemology) (Kivunja & Kuyini, 2017). The study is also theoretically informed by Figley’s (1995) foundational conceptualization of secondary traumatic stress, Stamm’s (2010) Professional Quality of Life (ProQOL) model, and DSM-5 criteria for secondary traumatic exposure (American Psychiatric Association, 2013). These theoretical conceptualizations and models shaped the research questions and provided an interpretive lens, alongside participant co-constructed meaning-making, thereby aligning with RTA’s allowance for theoretically informed deductive elements within a predominantly inductive analytic approach (Braun & Clarke, 2022).

We continuously engaged in a reflective process throughout the study and recognized our positionality as researchers as essential to the study. A separate section on researcher reflexivity and positionality is provided as an online supplementary section (Researcher Positionality and Reflexivity). Additionally, fieldwork notes and reflexive journal excerpts provide an audit trail in the online supplementary materials (Figure S1, Reflexive Journal and Notes).

The specific research questions guiding the study are:

- What are the experiences of STS in school personnel working in non-higher education institutions (secondary level and below) in Ireland and the U.K.?
- How do school personnel deal with any effects of secondary trauma and perceive sources of support when dealing with secondary trauma in the school environment?

## Methodology

### Design

We employed a qualitative design, using individual semi-structured interviews via online video calls. We used Reflexive Thematic Analysis (RTA) as the methodology due to its theoretical flexibility and its fit for addressing research questions about experiences, perceptions, and people’s practices and behaviors (Braun & Clarke, 2022). Reporting was guided by the Reflexive Thematic Analysis Reporting Guidelines (RTARG; Braun & Clarke, 2024).

### Participants

Recruitment initially involved systematic email outreach to formal gatekeepers at randomly selected schools from U.K. and Ireland databases. Due to insufficient responses, convenience and snowball sampling, along with social media outreach, were used to recruit participants. Eligibility required individuals to be current or former school staff in a teaching or support role for at least one year, have awareness of a pupil’s trauma, be over 18, and be able to give informed consent in English.

The final participant group consisted of 11 school personnel from Ireland, Northern Ireland, and England. Of these, six were currently based in NI, four in England, and one in the RoI; though one of the England-based participants had migrated from the RoI to England and recounted experiences across both contexts. A summary of participant pseudonyms, region/country, and years of experience is available in Table 1 (Appendix A). Purposive sampling, targeting subpopulations with particular knowledge or experience with the phenomenon (Palinkas et al., 2015), was used to ensure participants had the necessary exposure to answer the research questions and to capture a range of school environments and roles. The recruitment strategy included personnel with varied years of experience from primary and secondary schools of different sizes and socioeconomic contexts. As a token of appreciation for participants’ time, they were offered £5 to donate towards an educator charity, a practice which evidence suggests can improve response rates (Abdelazeem et al., 2022). Participants were also offered entry to a random drawing for a £50 gift voucher from a popular online retailer.

The sample consisted of three males and eight females, mostly aged 29–39 (70%), with two over 39 and one under 29 (one did not answer this question on the demographic proforma). While some participants held overlapping roles, the main job roles included teaching, pastoral support and safeguarding (student-facing well-being and guidance functions broadly comparable to a school counselor), special education, and principals/heads of school. Experience ranged from 3 to over 20 years. In terms of school setting, six participants worked in secondary schools, four in primary schools, and one worked across both. The context of participants’ schools was also varied: some recounted experiences from both publicly funded and private school settings.

The final dataset size of 11 was guided by the principle of information power, which suggests that sample adequacy primarily depends on the study''s aim, although population specificity, theoretical background, quality of dialogue, and analysis strategy play a role (Malterud et al., 2016). The decision to cease recruitment after 11 participants was made during data collection and initial analysis, once it was determined that the data quality and depth were sufficient to address the research aims.

### Procedure and Data Generation

Following institutional ethical approval, the recruitment materials and semi-structured interview schedule were piloted with two former secondary teachers from Northern Ireland, with over 20 years combined experience, who contributed solely to instrument refinement and were not part of the study sample. The “Think Aloud” method was used during this phase, in which the two former teachers worked through the interview schedule of questions and recruitment materials, providing immediate feedback to the primary author via a verbal thought stream (Eccles & Arsal, 2017). Their feedback then led to refinements in the wording of the schedule, the demographic proforma, and the recruitment materials (online supplementary materials, Figure S2 and Figure S3).

The primary author conducted all 11 interviews via video call from January to August 2025. Interested individuals who responded to recruitment materials were provided with a detailed information sheet that included the study''s purpose and completed an informed consent form before data collection. The semi-structured online interviews lasted approximately 60 min and were audio-recorded for verbatim transcription. This online modality was chosen for its resource benefits for both the researcher and participants (Carter et al., 2021). Per institutional ethical guidelines for potentially distressing topics, the primary author debriefed each participant after their interview, signposting local mental health resources and invited final feedback. Transcription and anonymizing data were conducted by the primary author, with software assistance from Whisper Transcribe (v. 1.4).

### Ethical Considerations

Institutional ethical approval was obtained from [Removed for blind review] before any recruitment or data generation commenced. The procedures used in this study adhere to the tenets of the Declaration of Helsinki. Participation in the study was entirely voluntary. Furthermore, participants were made aware of potential risks through the study information sheet and informed of their right to withdraw. [Ethical approval document is available in a cover letter to the Editors].

### Data Analysis

Data were analyzed using Reflexive Thematic Analysis (RTA), according to the iterative steps outlined by Braun and Clarke (2022). The process was predominantly inductive, with codes and themes actively constructed from the bottom up and grounded in participants'' verbatim accounts. However, some aspects of the analysis were theoretically informed in a deductive manner. Specifically, research questions and some interpretations of narratives within themes were shaped by existing literature, including, but not limited to, STS models, as described earlier. The complete analytic and coding process is described narratively and tabularized in the online supplementary materials (Figure S4, Data Analysis and Coding). Additionally, the Theme Map is illustrated in Figure S5 (Thematic Map).

## Analysis

We created four main themes to represent the shared patterns of co-constructed meaning across the stories of 11 participants. While the themes are anchored in the convergent accounts of the participant group, key divergent cases are integrated to capture unique perspectives and nuance. The following narrative analysis presents these themes using pseudonyms and verbatim extracts from the participants. The themes are: (1) *A System Under Strain: Institutional Failures and Inadequate Support*; (2) *The Intrusive Weight of Trauma: An Emotional and Personal Toll*; (3) *The Nurturer and The Fixer: Negotiating Professional Identity vs. Personal Responsibility*; and (4) *Navigating the Burden: A Patchwork of Coping Mechanisms and Possible Protective Factors.*

### A System Under Strain: Institutional Failures and Inadequate Support

What participants recounted was not a set of discrete, detached stressors but rather an inseparable system-level experience, comprising conditions that seemed to compound one another, creating an environment in which STS could take hold. Workload and time pressure, the performative nature of formal training and support, the absence of meaningful supervision, and a climate that discouraged help-seeking are examined below, and they are best understood as mutually reinforcing features of the same environment theme rather than independent problems.

This theme describes participants'' shared experience of working within a system that appears to fail to adequately support them. A ubiquitous narrative was that of workload pressures and time constraints, with Dana stating, “It''s very bogged down, everyone''s very busy...”, and Barbara stating, “There isn''t actually time in our jobs for us to take that time to, you know, like decompress”. Data from consistent participant sentiments suggest that the strain might be exacerbated by institutional and systemic priorities that focus on academic performance metrics over staff and student well-being. Lee illustrated his experience with this, “…there was an inflexibility to listen from management. I think the pressures from Ofsted [Office for Standards in Education] and performing were such that they just felt like, you just got to get it done. You know, you just gotta”. This imbalance was a central point of contention for school staff, as William shared with frustration:

. . .it should not be 90 [%] academic and 10% how to be a person, you know, it needs to be right, closer to 50/50. . . that brings me into conflict with other colleagues and the principal…

Formal support and training were consistently described as performative or "tick-box" exercises, a perception captured by the constituent code *Perceived Inauthenticity of Formal Support*. Lee suggested that time pressures made such training ineffective:

. . .you know, there''s training, tick box training. You share a presentation for an hour, . . .I don''t have those two hours that getting to grips with the basics of what was just said will take.

This perception of performative support was reinforced by Kelly, who described a one-off meeting on trauma-informed practice that was never mentioned again and felt "tone deaf" to the potential trauma histories of the staff in attendance:

We had a one-hour meeting about it and then it was never mentioned again. There’s no policy. . . it was like they just needed something to kind of fill this hour. . . the whole thing is a little bit tone deaf because …the likelihood is that there are people in there who are experiencing trauma.

There was a consensus among the group that they felt unprepared by their initial training for the socio-emotional realities of the role, a sentiment illustrated by Bonnie:

I know how to teach long multiplication, and I know how to teach the difference between a noun and verb, but that whole social and emotional piece is somewhat airy fairy. . . I’d say we had no preparation for working with children who were coming from difficult situations at home.

The lack of genuine support was particularly critical regarding supervision, with a common sentiment being, “we don’t have supervision... and I think we should” (William). Ashley’s experience summarized a prevalent feeling of frustration with inconsistent, “tick-box” check-ins:

I am not in receipt of supervision yet. I have been pressing for it for a while now. My line manager is extremely busy. . . We''ve tried to do like a schedule. . . [and when they do meet] it''s not really giving me what I need. . .It''s more like a tick box that she''s seen me.

The absence of meaningful supervision was not a singular gap; it operated within, and potentially reinforced, a broader cultural climate that seemed to discourage asking for help. Participants also felt that institutional shame-based cultures and language impacted their support-seeking and well-being. Lee discussed a "shame-based" culture that discouraged help-seeking:

. . .the culture is still kind of shame-based. So if you''re not doing well in your behavior management, then whoa, you know there''s something wrong with you. . .I don''t know if I want to sit too close to you or stand too near to you because I might catch whatever you''ve got. . .

Claire critiqued how terms like "burnout" and "resilience" were instrumentalized and used to place responsibility on the individual rather than “the system”:

. . .there''s all this talk of, like. . .To manage burnout, you have to do whatever for yourself. . . It''s individual-centered. It''s not systemic. It''s an individual burns out. The system doesn''t burn you out. It really makes me angry… [Resilience is] a really awful HR word. Masculine word. . . [Empathy] It''s a female word, I think. I think it''s associated with women. . .I think it''s misused a lot. . . it''s kind of wrapped up in this whole “be kind” thing. . .Which, for me, seems performative.

The use of instrumentalized language, “tick-box” initiatives, and inadequate support with an anti-support-seeking culture can leave school staff feeling overwhelmed by the effects of secondary trauma. The current analysis shows that staff who work in high-exposure roles, such as safeguarding and pastoral leadership, can be particularly vulnerable, as Barbara, a special education leader with designated safeguarding duties, states:

You can just feel like you''re surrounded by negativity and it''s just all the time and you''re like…I would [like to be] a resilient person. But you just. There are days you just think, I can''t listen to any more negativity.

### The Intrusive Weight of Trauma: An Emotional and Personal Toll

This theme captures the personal cost of secondary trauma exposure that results from working within such a strained system. This "weight" or feeling “bogged down” can manifest as specific, symptom-like experiences that recur across participants’ accounts and permeate their personal lives. This burden is concisely summarized by Barbara’s statement about the impact of her pastoral responsibilities, “...[it] can weigh on you”. The theme is constituted by codes such as *Intrusion into Home and Personal Life* and *Intrusive Traumatic Imagery*. William, who recently left a pastoral role, described how details from a parent’s disclosure resulted in persistent mental images:

. . .those images gone through my head that the, the, the mom had so graphically described were deeply disturbing and I couldn''t, I could not shake those images out of my head. It was like I was watching Hammer Horror films in my head. . .I couldn''t get rid of them.

The toll was often felt physically. After reading a disturbing report with another safeguarding colleague, Barbara recounted, “...[it] did actually make us both feel sick…”. This physical impact was also exemplified by Claire, whose own past trauma was reactivated after learning a student’s father had died similarly to her own father''s medical crisis:

. . .for myself, I had a really strong, like, emotional, physical, emotional response that I remembered from when my own father had a heart attack. . . It was a very similar feeling. I recognized the feeling, actually.

Claire also provided specific descriptions of the effects she had experienced, “it''s a huge physical drain on you and the night... I found it really hard to sleep as well”.

Participants'' experiences also appeared to be shaped by a relational identification with students, such that staff saw their past selves in their students. This “mirrored” trauma, as Ashley termed it, could be used to build an empathetic connection:

I often see this kind of mirror, mirrored thing happening here. . . because her particular experience, it wasn''t exactly the same, but I grew up in a household where there was a lot of volatility and arguing… I did say to her, I know what that feels like to be in a house where there''s screaming, shouting, and you feel trapped. So I see you, and I''m sorry that you have to go through that.

However, overly identifying or connecting with past childhood roles may also trigger traumatic stress responses in staff, as Ashley states, “...it''s being the one to be calm [during a chaotic home visit]. That part was triggering for me...I''ve done that as a child...it just, it was like, damn, I''m really, I''m this person."

The emotional toll of secondary trauma also appeared to alter participants’ interactions with the broader student body. Kelly described becoming impatient and “cross” with other students after learning of one student’s significant trauma:

I think more at the time it can actually be interestingly kind of affect your patience with other kids. . .I felt at the time . . . almost like cross with the other kids if they were rude or did something wrong, because it''s like you''ve got everything great for you. . . like you don''t have to go through, what that boy is going through. . . .

The data also suggested that effects of exposure to secondary trauma could be long-lasting, remaining with staff after the initial exposure for up to years and intruding into their personal and home lives. These effects were stated in narratives of worry, guilt, mood disruptions, isolation, and sleep disturbances at home. Several participants illustrated this: “the sadness, the loneliness, the isolation, the frustration. My sleep hasn''t been great” (William), and “...when I work with particular young people, it affects my mood and my own emotions when I come home. Because you are left holding their despair. It''s hard to sit with” (Rochelle). Furthermore, Maggie shared how worry caused her to behave:

You would go home and you would worry. I, I would take it home. I would worry about it. I would, you know. . .If you were driving past the area where your school is, I might detour to see if any of my wee ones are out and about.

Likewise, the disruptive effects of secondary trauma exposure can intrude on staff’s relationships at home, as William explicitly states, “… it was creeping into my home life too much... a lot of stories that you were listening to were moving around my head a lot while I''m trying to be present for my then 4-year-old.” School staff seem to experience guilt based on three foundational narratives: retrospective guilt; “I nearly feel bad now that I didn''t feel worse” (Bonnie), guilt from comparison between the trauma-exposed student and one’s own life and children; “...looking into my child''s eyes and, and thinking why can''t other children have what you have?” (William), and guilt from not being able to help or not knowing sooner; “I feel bad that I hadn''t...known about it previously” (Dana). Kelly, a secondary school teacher, also described how several years after learning of a past student’s potentially traumatic experience, and no longer working in that school, there remains not just thoughts, but a physical effect stemming from the guilt of leaving without being able to help:

That was 10 years ago. . .I have often cried over that boy because I think, like, oh, if I hadn''t, like, I left kind of in his. . . last two years of school. . .it can still have quite a physical effect on me, like remembering that, that horrible event. . . there''s definitely still like almost a physical kind of response to it rather than just thinking about it. You know, I still find it physically upsetting.

Combined, the weight of the emotional and personal toll that school personnel feel from interacting with students who experience trauma, knowing about their specific traumatic events, and the performance demands of teaching can feel overwhelming, driving staff to leave their roles. The current analysis suggests that roles with leadership and safeguarding duties may be more vulnerable to attrition from the emotional toll, since, as Bonnie recounts, these roles expose staff to “a world that if you were a normal teacher, you wouldn''t know existed”. Bonnie stated further, “Actually, our vice principal…stepped away from the safeguarding role because he couldn’t absorb…so much of it, coming at them”. And Lee, a secondary teacher who relinquished his pastoral role due to unsustainable demands, summarizes it as, “it just became too much.”

### The Nurturer and The Fixer: Negotiating Professional Identity vs. Personal Responsibility

This theme, consisting of statements like, “My job was, my whole life”, by Kelly, explores how professional identity might shape the experience of secondary trauma. Staff’s identities are often entangled with a personal sense of responsibility that blurs professional boundaries, manifesting as ‘Nurturer’ (a parental, frequently mother figure) and ‘Fixer’ identities. Furthermore, this theme explores how negotiating these identities can impact personnel''s feelings of satisfaction from making a difference or distress from their limitations in helping. This theme also captures how identities are not static, but rather are negotiated and can shift over the course of a career due to life events.

The level of responsibility that school staff feel towards their duties and organizations seems to influence how they obtain and engage with their roles. As Bonnie suggested, the safeguarding role that she assumed, in addition to already being a principal, was taken because of a feeling of involuntary obligation, “...nobody else wanted to move, so I moved”. Temporary or substitute staff, such as Matt, have a different perspective on their responsibility:

. . .you could have been in the school for maybe just two days. So like you''re not really like thinking about it. . .It''s sort of like a half-eight, or 8:30 to 3:30. You''re sort of zoned in and then once that, once that 3:30 came you''re out.

Conversely, Maggie, a primary school teacher in a permanent role, expressed an identity that was consistent with a nurturer and parental figure: “I always see myself as their mother in school. They are little babies, you know... they need that figure in school.” There can be a blurring of identities and personal responsibilities, where teachers extend themselves beyond the classroom. Maggie later provided an example of this blurring and stated how it was “part of the job”:

. . .buying extra food for the classroom, stocking up on cereal bars. . .going out and buyin'' a pair of shoes or socks or whatever. . .it''s all part of the job, kind of dealing with those behaviors, embracing those behaviors and solving them.

This nurturing or fixing drive could lead to positive feelings when staff felt they were “making some sort of difference… in a world of inertia” (William), but could also result in "heartbreak" and feeling "devastated" when they were powerless to help. Kelly’s reaction to one such case reflected this through the parental identity: "...in that case, I was absolutely heartbroken... It''s awful if there''s nothing you can do to help… So it was like it was my own child". Barbara’s recollection of a conversation with a colleague lends depth to the "fixer" narrative by explicitly referring to herself as such; “...she [a colleague] actually had said to me, ‘but sometimes there actually isn''t an answer’ because I would be a fixer...She was like, ‘there is no fix’. I couldn''t deal with that”.

Some staff, like Barbara, may experience distress from being unable to change students’ trauma-related circumstances and outcomes. However, others appeared to negotiate their identities and responsibilities to help or fix with more separation. Ashley, who had previously shared some active coping methods, stated that she realizes that there are times when there was nothing she could do but hold “space”:

. . .and one thing that I''ve realized is sometimes you don''t need to give a solution. That''s not what you''re there for. You just need to hold space. And so I just listened. And it''s hard. That''s hard for me. But I just held space because there''s nothing I can do.

As demonstrated through participants'' own words, school personnel’s professional identities are intertwined with their sense of personal responsibility and may influence how they navigate and experience the effects of secondary trauma exposure in their roles. However, participants'' professional identities were not static. The data showed that identity is negotiated over time, and some staff, like Ashley, can develop an adaptive perspective. Furthermore, major life events could prompt a significant shift in priorities. Kelly described how becoming a parent created a healthy detachment from a previously all-consuming professional identity:

. . .for like 15 years. . . my job was, my whole life, my whole identity was wrapped up in being a teacher. . . Whereas then when you become a parent that changes and that''s not who you are anymore. It''s just what you do. . .

## Navigating the Burden: A Patchwork of Coping Mechanisms and Possible Protective Factors

The current theme mainly answers the second research question and captures how school personnel manage the effects of secondary trauma in their roles. In the absence of adequate institutional support, staff appeared to rely on a patchwork of coping strategies. The most valued form of support came from informal and relational interactions with colleagues who were perceived to have shared experiences. As William stated, “just unloading…it made you feel better,” and talking with other staff who were “all in the same boat” allowed him to cope with the burden, “knowing that we’re all dealing with it”. Even when basic telephone access to counseling is provided to staff, they often do not value this type of support as much as collegial support, and perceive it to be just as performative as the training, as Bonnie suggests:

. . .we have access to counseling and stuff like that if we need it, but I do feel like that''s just to tick the box. . . Personally myself, if I was going through something, I''d be more likely to go to my own GP. . .than through. . .this other setup.

Claire also supported this idea, that a shared connection with peers, when sincere and non-intrusive, can potentially mitigate some effects from difficult disclosures about students’ trauma:

One of my line managers. . .I think she and I had a kind of similar view of it. . . she would just check in with me and I would check in with her. And I found that really helpful, actually, because it was not intrusive. It was not like tokenistic either… actually, she messaged me a few times… it was that kind of acknowledgment of, like, I''m sure this is on your mind. And so it was. That was really good, actually.

However, confidentiality demands sometimes limited peer connection with non-privileged staff, as Barbara stated, “...you usually can''t share what it is [with other staff]”. This restriction sometimes prompts staff with limited peer support options like Ashley, who works within a “team, [that] doesn’t feel like a team”, to pretend to be “fine” when interacting with non-safeguarding colleagues: “...because of my role and because I work with the safeguarding team, I can''t really say anything. So it''s like, yeah, I''m fine. But I''m not fine. I''m not. Had a hard day. It is what it is”. Conversely, other staff experience the potentially STS-mitigating benefits that come from connecting with a team of equally privileged peers. Barbara, who can share experiences with a team of four safeguarding staff, states, “I think being a part of a team really helps...having the team helps you to manage it”.

Participants in the current study presented a range of perceived severity and acknowledgment of the impact of secondary trauma, which may be indicative of their coping strategies or protective factors. One participant, Matt, a former substitute teacher who admittedly was rarely exposed to secondary trauma, and never directly from a student, stated, “not really sure if it affected myself outside of work or anything”. Bonnie suggested that the impact of a specific disclosure was modest; "truthfully, I would say nothing that, you know, nothing that left me sleepless or anything like that”. Furthermore, Dana, the youngest participant who had been teaching for approximately three and a half years, suggested that the impact of her secondary trauma exposure on work or home life was unnoticeable, being somewhat numb to any changes due to secondary trauma:

. . .if I''m going to be honest, this year has been quite stressful anyway. And so I didn''t really notice, like, any stress changes. It''s just been a general stress. So it''s kind of like, oh, it''s another thing on. . .so. No, no, it didn''t. Which is really sad to say. . .

Participants also described using compartmentalization as a coping strategy; as Bonnie later explained, “I probably did put it in a box, you know, and… you move on with the business at hand”. Others engaged in more intentional self-care. Ashley, who emphasized “I get my own room”, noted, “I''ll put on my jazz and I''ll turn off the light and I''ll sit in the window and I''ll be okay...”. Ashley provided a detailed account of her proactive coping approach:

I keep my journal in my bag, so I just, I write it down. . .I''ve definitely made peace with my experience of childhood, which is why I feel that I am okay in this role, even on the hard days. So although I, I replay it in my mind, [pause] I send a lot of love to my inner child. Like a lot of love. A lot of love [emphasis]. And so that''s why I feel equipped to do it…I''ll be honest, since I started this role, I have gone back to therapy just because. For a few reasons, but definitely… Because I wasn''t getting supervision, I just wanted to make sure that I can be healthy.

However, even with proactive self-care steps, Ashley also admitted that sometimes the lack of supervision and the “expectation to just get back to work” drove her to cope in less-than-ideal ways, such as alcohol use:

. . .but it was still a lot. . .To the point where. . . I went to the pub after work… that day I did go to the pub and have a couple glasses of wine. But…It''s not my preferred method to handle a stress day.

Although one participant stated, “I think it''s very hard for me to find positives...” (Dana), most school personnel relayed some positive effects of supporting trauma-exposed students. For example, Maggie, a primary grade one teacher, expressed, “You know, it''s really magical to see how much you can get out of a wee one. They''re just sponges, but honestly, it''s. It is very, very positive”. And William stated, “I wouldn''t call it satisfaction, but I would call it...you know, that you felt you were making some sort of difference... in a world of inertia”.

One participant''s experience of navigating secondary trauma was in stark contrast to the others, and highlighted the benefits of effective, multi-layered formal support. Rochelle, who worked in schools but for an external agency, was provided with formal supervision and protected time for self-care, a situation that opposed the experience of all other participants:

We are very lucky because we have supervision. . .we talk about sort of vicarious trauma and secondary trauma and what we can do to help ourselves. . .we have like a well being hour that we get to take once a week. . . So I feel lucky in the team that I''m in because they do think about the impact of the work.

Rochelle also stated, “I suppose sometimes it makes me really in awe of how resilient some of the children are...I do sometimes wonder, if that was me in that position, would I have had the strength to go on,” which reflects a unique perspective of experiencing resilience through her students. The absence of such formal support, as offered to Rochelle, led others to call for more proactive measures from their own school leadership. Participants expressed a desire to move beyond a reactive stance, with Claire stating, “everything is always reactive”, and proposing a structured, preventative approach:

We spend all this time thinking about how to care for the kids. . .And we will have meeting after meeting and... very little consideration goes into how to care for the adults. I think that just, like, they have risk assessments for, like, if a bomb went off in the school and you have your, like, “gold team” that''s there to, you know, deal with the situation… I think there should be, like, an emotional equivalent of that.

## Discussion

This study explored the experiences of secondary trauma and, potentially, secondary traumatic stress (STS) among school personnel in Ireland and the U.K. Through co-constructed meaning between participants and researchers, we produced an analysis of four key themes that illuminate the professional and personal toll of this work, along with the systemic, relational, and identity-based factors that influence these experiences.

### School Personnel’s Experience of STS: A Symptom Profile

The analysis offers a rich, experiential account of how school personnel encounter and navigate secondary trauma. While one participant (Rochelle) felt adequately supported by their organization and “lucky” to work in a team that considered the impact of trauma in their role, others felt inadequately supported by their organization and underprepared to work with traumatized students through “tick box” initiatives. All had been exposed to information about students’ adverse circumstances and shared a range of effects. Consistent with RTARG by Braun and Clarke (2024), while maintaining a constructivist view, these effects are contextualized through the lens of existing STS theory to further the discussion.

This discussion frames symptoms and effects through a qualitative lens rather than a diagnostic one. While clinical criteria inform the analysis and are used for comparison, the study does not intend to assess the duration or symptomatic clusters necessary for formal diagnosis. Instead, by asking participants to reflect on their careers holistically, the research captures the qualitative range of STS as an experienced phenomenon. This deliberate choice shifts the focus from clinical status to the subjective meaning school personnel assign to indirect trauma within the systems they navigate.

The effects of secondary trauma exposure were generally described as an emotional weight, with varying degrees of feelings of initial sadness, anger, and guilt. Some participant accounts reflect symptoms of STS that align with existing clinical and theoretical frameworks. William’s description of unshakeable “Hammer Horror” images “gone through my head” parallels the PTSD diagnostic requirement of intrusion, including re-experiencing the trauma, in both the DSM-5 and the International Classification of Diseases 11th Revision (ICD-11). Furthermore, his narrative of stories “moving around my head” might also be interpreted as rumination, which literature suggests is “significantly and strongly correlated” with STS (Fraser et al., 2024, p. 117). We also found that some school staff in this participant group openly worry about their students with known adverse circumstances. This worry can extend beyond the classroom, as staff like Maggie would “take it home”, sometimes driving through the school community to check on the “wee ones” outside of regular work hours. This suggests that intrusive worrying may not just be internalized but may prompt behaviors potentially aimed at soothing distress. Experiential data from this study also aligns with elements of DSM-5 criterion E, demonstrating that intrusive thoughts can impact sleep, such as Claire, who “found it hard to sleep,” and William, whose “sleep hasn’t been great.”

Beyond intrusion, the description by Kelly of feeling "cross" with other students, after learning about the trauma of another, potentially embodies an increased level of irritability linked to PTSD and STS arousal or mood changes (Dominguez-Gomez & Rutledge, 2009; Fleckman et al., 2022; Sprang et al., 2025). A connection between Kelly’s increased irritation and perhaps their altered perceptions that other students were doing “something wrong” has been supported by literature suggesting that as teachers’ self-reported STS symptoms increased, so did their ratings of students’ socio-emotional behavior issues in the classroom (Simon et al., 2022). We posit that physical indicators of mood changes, such as crying, and other somatic representations, such as feeling “sick,” may occur in staff due to secondary trauma exposure. Staff’s arousal changes can manifest as irritability towards other students, which could influence disciplinary actions against students and overall interpersonal relationships within the school.

While effects aligning with intrusion and mood/arousal changes were clearly represented in the data, one category was markedly underrepresented. We noticed that the data from this participant group did not show evidence of the strictly defined revision of avoidance criteria in DSM-5 and STSS. This is important because “avoidance” of trauma-related reminders is a required characteristic for PTSD diagnosis (APA, 2013) and constitutes key subscales of the STSS and ProQol measures (Bride, 2013; Bride et al., 2004; Stamm, 2010). We can concede that, on the surface, Ashley having “a couple glasses of wine” or Bonnie putting thoughts “in a box” to focus on the “business at hand” could be interpreted as avoidant behaviors in the moment. However, no participant stated, either semantically or latently implied, that their thoughts or behaviors were aimed at avoiding thoughts, people, or reminders of the secondary trauma exposure. Moreover, the co-constructed meaning was not one of escape, but of social peer connection in Ashley’s case of going “to the pub”, and practical compartmentalization in Bonnie’s case. The DSM-5 criteria and STSS subscale changes, removing items and making “avoidance” more oriented to actively avoiding people, places, or, in this case, students, rather than passively being numb, support the limited presence of avoidance in this group. Similar findings were reported by Fleckman and colleagues (2022, p. 945), who stated that “avoidance… was not prominently identified” among their participants, public-school educators. Moreover, the study suggested that, given the nature of their job roles, this finding may indicate that school staff are less able to use avoidance (Fleckman et al., 2022). This may well have implications for how STS is evaluated in this population.

Not all school staff will reveal effects or attribute their symptoms to secondary trauma exposure. Personnel like Matt, who was “not really sure,” or Bonnie, who said “nothing that left me sleepless or anything,” could indicate levels of unawareness and symptom minimization. While we cannot definitively state whether this is due to protective factors or infer any other causal relationships from this study, literature suggests that educators may lack metacognitive awareness or deny effects due to the “grandiosity of their role” (Essary et al., 2020, p. 118). Consequently, organizations must adequately educate staff to identify potential effects. Although Dana’s statement of “I didn’t really notice any stress changes” illustrated that some staff operate under “general stress”, it may also imply a level of numbness or detachment, symptoms of STS now in the ‘cognitive changes’ domain (Frearson & Duncan, 2024). However, Ludick and Figley (2017) propose that detachment is part of a resilience model (CFR) against STS, which makes this an important point to explore in the resilience discussion section.

#### The Relational Nature of Secondary Trauma Symptoms

The analysis proposes that STS in this sample is deeply relational and influenced by how closely some staff relate their students’ circumstances to their own, whether currently or in the past. The idea of a shared or “mirrored” traumatic experience, as described by Ashley, has been referred to as “shared traumatic reality”, which can have both negative and positive outcomes (Dekel & Baum, 2010). Ashley did not appear to perceive this “mirrored” perspective negatively, but instead used it as a tool to show understanding: “I know what that feels like”. According to Dekel and Baum (2010; p. 1932), professionals having this shared experience with clients can help them respond “more empathetically and effectively in sessions”, which seems to be evidenced here. Nevertheless, absorbing others'' trauma in highly relational student support roles can be considerably challenging. Barbara’s admission of reaching a point where she cannot “listen to any more negativity” might suggest compassion fatigue, which Figley (2002) states “reduces our capacity or our interest in bearing the suffering of others” (p.1434). Additionally, re-experiencing trauma via “familiar” feelings, as Claire described, suggests that staff with their own trauma history may require special considerations, as they can potentially relive their own emotions when exposed to student trauma.

### The System as a Factor: Organizational Culture, Leadership, and the Conditions for Resilience

What participants describe in Theme 1 is beyond a simple lack of formal support. Instead, it demonstrates how "tick-box" training, inaccessible supervision, and unsustainable workloads converge with a culture that discourages help-seeking, actively weakening the conditions necessary for resilience. Viewed through Stamm’s (2010) Professional Quality of Life (ProQOL) model and an Ecological Systems framework, STS ceases to be a purely internal reaction to exposure. Rather, it functions as a phenomenon moderated and often exacerbated by the organizational environment. For many participants, the personal weight of secondary trauma was compounded by a workplace that failed to provide an adequate buffer against it.

Rochelle’s divergent account is significant, as it suggests that unsupportive organizational and leadership environments are not an inevitable feature of working in school contexts. From her perspective, her organization provided significant leadership and structural support, including a team-based approach to processing trauma exposure. This pattern shifts responsibility for resilience from the individual to the institutional level. The pattern aligns with Stamm’s (2010) model, which predicts that effective buffering against STS requires an environment that actively develops and sustains coping capacity rather than relying solely on individual effort. Claire’s observation, and somewhat satirical commentary, that "an individual burns out, the system doesn’t burn you out," captures this distinction with precision. These data suggest that without an organizationally supportive context, individual coping strategies, regardless of how well-intentioned, may prove insufficient to sustain well-being in high-exposure roles. Shifting the focus from how staff members manage STS toward how organizations reduce the structural conditions that amplify it represents a significant and necessary reframing within the current literature.

### Resilience, Coping, and Professional Identity

The findings highlight a complex relationship between professional identity, coping, and resilience. The ‘Fixer’ and ‘Nurturer’ identities, common among participants, are potentially foundational to a level of risk for STS in school staff. This risk stems from the motivation to improve the condition of a traumatized or suffering person (Figley, 1995, 2002). Furthermore, literature suggests that teachers and students often build an attachment, which sometimes causes teachers to respond to the “caregiving needs” of students like a parent (Kearns & Hart, 2017). Sentiments of powerlessness, heartbreak, and being “devastated” due to the inability to fix students’ circumstances were expressed in the narratives of staff who endorsed these identities. Social identity theory suggests that when valued professional identities are undermined, psychological risk increases, whereas maintained or developed identities serve as protective resources (Muldoon et al., 2019). For staff whose professional sense of self is rooted in relational care, persistent powerlessness in the face of student trauma represents a fundamental identity threat rather than mere emotional distress. This identity threat is compounded by institutional cultures that prioritize performative metrics over relational values (Skerritt, 2019). Consequently, many participants may face a dual vulnerability: the intrusive weight of secondary exposure and the erosion of the professional meaning.

Other staff, like Ashley, appeared to negotiate their identity and responsibility by learning to create some distance and "hold space" rather than trying to fix. This approach, combined with their intentional self-care, aligns with components of the Compassion Fatigue Resilience (CFR) model (Ludick & Figley, 2017), which was introduced earlier. The identity of “Nurturer/Parent” can also bring a duality of experiences, depending on how much detachment and perspective-shifting are used. On one hand, staff like Kelly can be “absolutely heartbroken” at the inability to help a student when it felt “like it was my own child”, then also shift professional identities from “my whole life…being a teacher” to “it’s just what I do”, after becoming a parent herself. This shift in identity and perspective appeared to provide Kelly with some disengagement from her previous, all-consuming teacher identity. Staff may potentially experience a shift in the opposite direction, where, like Rochelle, they find it “more difficult after I had my own biological child...especially whenever there''s a similar threat”. In this scenario, it appears that staff may find it more difficult to disengage from the parent identity due to the similarity of plausible trauma threats to their biological child.

Detachment, introduced earlier as a potential symptom of STS that was reflected in some participant narratives, seems to be a complex construct that can either serve as an adaptive psychological coping mechanism for STS or a maladaptive one. Extant literature suggests that detachment is associated with lower STS in school personnel (Phillips & Anderson, 2025) and that the ability to “let go” of the suffering of clients is a significant component of the CFR model (Ludick & Figley, 2017). However, detachment is thought to only temporarily distract individuals from the guilt-related effects of self-reported traumatic symptoms and can prevent recovery from PTSD symptoms if not adequately processed (Held et al., 2011). Thus, school staff who use detachment and compartmentalization as short-term, goal-oriented coping, while integrating self-care, social support, and other intentional forms of processing secondary trauma exposure, may develop stronger resilience to STS. Conversely, staff who only rely on detachment, without actively processing their vicarious trauma exposure, may suffer long-term effects, potentially driving them to leave the role.

While the negative impacts were significant, the data also led us to recognize some positive experiences from staff interacting with trauma-exposed students. It was common among participants in this group to express good feelings from making a “difference”, whether in their teaching or student support roles. Interestingly, although William stated that he would not describe his positive experience of making a difference as “satisfaction”, literature suggests that compassion satisfaction (CS) deals with a practitioner’s “sense of self-efficacy” related to their work (Oberg et al., 2023; p. 3), such as making a difference. This suggests that promoting staff’s positive perception of self-efficacy and affirming their contributions to making a “difference” might contribute to their compassion satisfaction. What is more, Rochelle’s unique experience of being in "awe of how resilient some of the children are" could reflect vicarious resilience (VR), which is characterized as a positive, transformational inner experience obtained by recognizing “trauma survivors’ own resiliency” (Hernández et al., 2007; p.237). Similarly, Rochelle’s statement could also reflect a pathway to vicarious post-traumatic growth (VPTG), which literature suggests is positively correlated with working in a high trauma-focused environment (Cleary et al., 2023) and predicted by active coping strategies and instrumental support (Kalaitzaki et al., 2023), which Rochelle felt “lucky” to receive from her agency.

Rochelle saw her organization as trauma-informed, stating they understand “the impact of the work”. Some researchers argue that aspects of trauma-informed education are too “deficit-focused” (Miller & Flint-Stipp, 2024). However, many other studies support the implementation of trauma-informed training and practices in schools, which benefit students, teachers, and the community (Brown et al., 2022; Brunzell et al., 2016, 2021; Castro Schepers et al., 2025; Christian-Brandt et al., 2020; Greig et al., 2021). In the current study, Rochelle offered an essential counter-narrative to the experiences of inadequate support shared by most other participants. Their agency’s multi-layered framework, comprising regular supervision, psychoeducation, and team-based collaboration, aligns with several of SAMHSA’s (2014) trauma-informed principles. Specifically, supervision and protected well-being time promote safety; team-based approaches foster peer support and mutuality; and active psychoeducation enhances transparency and empowerment. Notably, Rochelle’s account suggests that these principles, while often framed as student-facing, are equally applicable to organizational structures aiming to support staff. Although no causal conclusions can be drawn, their narrative serves as a convincing example of what adequate, trauma-informed support might look like, and that this type of support can potentially contribute to VR and VPTG in school staff. Overall, this study suggests that for some staff, without a multi-layered approach to staff support, the strained school system can become fertile ground for STS to exert its intrusive weight.

### Limitations

While this study offers an understanding of how personnel from a range of school environments in the U.K. and Ireland experience and manage secondary trauma, the narratives are specific to this sample. Therefore, the findings may not be generalizable to a broader population, which is a limitation. However, the breadth of participants'' roles and backgrounds, and the depth of convergent data, suggest a level of transferability to similar populations. Additionally, despite efforts to mitigate bias through rapport building, the potential for social desirability bias remains a consideration in interpreting these findings (Bergen & Labonté, 2020). A further limitation concerns the potential jurisdictional variability within the group. Although participants were recruited from the Republic of Ireland, Northern Ireland, and England, they were analyzed as a single group to ensure thematic coherence. The distinct accountability cultures and historical legacies across these jurisdictions represent a degree of contextual variability, a potential limitation that the current sample size and aims did not permit investigating in depth.

Future research should explore the “mirrored trauma” hypothesis to understand the extent to which shared trauma histories between staff and students influence STS outcomes. Furthermore, jurisdiction-specific studies drawing on larger, adequately powered samples within each context would be better positioned to examine whether STS profiles and help-seeking behaviors differ meaningfully across these environments, particularly regarding how distinct governance structures might moderate outcomes. Additionally, comparing the qualitative findings of the divergent case (Rochelle) with quantitative data could offer valuable insight. Specifically, research should examine STS and compassion satisfaction outcomes by comparing staff who receive multi-level organizational support with those who do not, thereby helping inform an appropriate model of support.

### Implications for Research and Practice

This study provides an experience-centered account of STS in an under-researched jurisdictional combination, framing it as an inherent occupational risk that may challenge standard diagnostic frameworks. The under-representation of avoidance symptoms aligns with evidence (Fleckman et al., 2022) suggesting a need to re-evaluate school-based STS assessment, while the concept of ‘mirrored trauma’ offers a lens for understanding how staff identities and histories filter student distress. For practice, school organization leaders should move beyond reactive, individualized well-being models toward proactive, structural supports such as protected supervision, team debriefing, and integrated psychoeducation. Rochelle’s experiential alignment with SAMHSA’s (2014) principles demonstrates that these supports are operational realities in some organizations rather than aspirational ideals, yet highlights that her ordinary infrastructure remains exceptional for most participants.

At the policy level, well-being provision across Ireland, Northern Ireland, and England requires structural embedding to bridge the gap between nominal requirements and lived experience. Regardless of governance and jurisdiction differences, reactive models appear insufficient to address the sustained, relational demands of supporting trauma-exposed students. Establishing minimum standards for supervision and processing time offers a more robust foundation than relying on institutional goodwill or individual resilience. Ultimately, these findings provide a qualitative basis for fostering a culture of systemic care and shared responsibility in educational practice.

## Conclusion

This study highlights the experiences of school personnel dealing with secondary traumatic stress in Ireland and the U.K. The findings indicate a consistent view, from this participant group, of a dissonance between systemic performance expectations and perceived unsupportive conditions, which potentially worsens the impact of secondary trauma. The personal toll of educating trauma-exposed youth can be significant and long-lasting, often intruding into staff members’ personal lives. The stories of the group in this study illustrate that some staff members carry their students’ despair home, have their past traumas reactivate at work, and feel powerless within their systems. The adverse effects of secondary trauma exposure likely vary based on several factors. However, in this specific sample, many participants attributed their effects to environments perceived as unsupportive, where performative gestures replaced meaningful care. Despite these challenges, school personnel demonstrated resilience by adopting various coping strategies and seeking informal support where possible.

## Supplementary Information

Supplementary Material 1 (DOCX 3.84 MB)

Supplementary Material 2 (DOCX 201 KB)

## Data Availability

The data that supports the findings of this study are available in the supplementary materials of this article and repository links.

## Appendix A

**Table 1:** Participant Summary

Pseudonym	Country/Region	School Level	Role(s)	Years of Experience
“Ashley”	England	Secondary	Pastoral/Safeguarding	~ 6 months in new role at the time of interview; 5 + years prior in related settings
“Barbara”	Northern Ireland	Secondary	Special Education Needs	18 years
“Bonnie”	Northern Ireland	Primary	Principal & Special Education Needs	22 years
“Claire”	England/Republic of Ireland	Primary at time of interview (Secondary in Ireland)	Teacher	~ 1 year (primary); 8 + years (secondary)
“Dana”	England	Primary	Teacher	3.5 years
“Kelly”	Republic of Ireland	Secondary	Teacher	16 + years
“Lee”	England	Secondary	Teacher; Pastoral	20 years
“Maggie”	Northern Ireland	Primary	Teacher	6 years
“Matt”	Northern Ireland	Secondary	Substitute Teacher (former)	4 years
“Rochelle”	Northern Ireland	Primary & Secondary	Student support (agency)	10 + years
“William”	Northern Ireland	Secondary	Pastoral/Safeguarding & Assistant Principal	7 years (pastoral) 14 + total

Table 1 summarizes participant pseudonyms, school level, general roles, and experience. Consistent with RTA reporting guidance (Braun & Clarke, 2024) on balancing participant anonymity with situating the sample, age, and gender, details are not included here and are reported only in aggregate in the manuscript (see Participants section) to reduce the risk of re-identification within this small sample. Roles are intentionally broad to reduce re-identification of participants with specialized or dual roles

## Declarations

### AI Statement

Audio from the interviews conducted in this study were transcribed with assistance from Whisper Transcribe (v1.4) and manually reviewed and corrected by the primary author for verbatim accuracy. Participants were made aware of its use in the information sheet and provided informed consent. Grammatical review and spelling were assisted with Grammarly (Superhuman Platform Inc) during the preparation of this manuscript.
