# Supplementary material for: “You Are Left Holding Their Despair”: A Reflexive Thematic Analysis of Secondary Trauma Among School Staff
Source: J Child Adolesc Trauma. 2026 Sep 9;19(4):2669–85. doi: 10.1007/s40653-026-00972-6 (PMC13737240; doi:10.1007/s40653-026-00972-6)
Supplement: Supplementary file 1 [file 40653_2026_972_MOESM1_ESM.pdf]

Supplementary Materials

Figure S1.

Reflexive Journal and Field Notes Excerpts

10/2024

***** *****

gov database were selected via this random selection method for the first phase of recruitment…

11/2024

After awaiting ethical approval for the amended materials, I resumed sending invitations to gatekeepers. Today I sent the next set of emails to 5 randomly selected schools in ***** *****

Journal Reflexive 1: December 2024

It is especially salient in the last weeks that I am learning some tough, and at times frustrating lessons about research. Having sent invitations to half of the first phase of schools and received no responses, it is now clear that recruitment via this very systematic method will be an uphill battle. School principals have a long list of priorities that do not include my research, at least not that they can easily conceive. Many schools also have boards and administrations who likely do not want their staff to air their job challenges. Therefore, it is no surprise that I have not received any responses, yet still disheartening. It may be time to change strategies.

Now I see why researchers opt for warm recruitment strategies, convenience, and snowball sampling. It is a matter of practicality. It is a matter of finding a solution to a problem.

1/2025

Final gatekeeper emails with recruitment materials sent today. ***** ***** I have received no replies. I really need to discuss commencing phase 2 and convenience sampling with the team.

2/ 2025

Amendments to the protocol were sent to ***** for approval of the sampling strategy change. This was granted. I could now use social media and more targeted convenience sampling, along with a small financial incentive.

Journal Reflexive 2: March 2025

After feeling like I would never get participants, I finally got my first participant, ***** *****

***** I was nervous, but prepared, as I had been practicing my interview schedule for some time. The first interview was with someone in …

I found that my initial nerves calmed down quickly, and I was able to connect very well with the participant. They were very talkative. Perhaps, partially because I was able to build

rapport, by letting them know that I too used to work in a similar role. They really opened up about their experiences and went into great details. In fact, the conversation flowed so well, that they were answering all my questions in so much depth, that I was already 45 minutes in and had only covered half the questions. I remember thinking 'how do I get them to stop talking so much', do I even do that? I was somewhat worried that I had told them the interview would be about an hour, and I would definitely need to go over this if I were to complete the full schedule…

3/2025

Things are slow going, but I'm starting to get some traction. I have 2 interviews under my belt and it is a good feeling to have data collection going in the right direction…

5/2025

Over the next few months of fieldwork, I was able to recruit and interview 8 total participants
*****
Although I have been considering the depth and quality of
is conducted and transcribed, now is a good time to really assess quality of the data and information power…

Journal Reflexive 3: May 2025

A large part of my motivation and perspective from which I conduct this research concerns my former role as a school staff member. I related deeply with many of the responses I received across all participants. This means that my interpretation and nuanced meaning making from responses is innately tied to MY own experiences within the school context. Consistent with RTA, I think this is a strong benefit that I bring to this research. Furthermore, my shared experiences with the participants allow me to understand the things that are NOT said. The silence, the sighs, the deep breaths between the things that ARE said, are also interpreted as meaningful. I remember this becoming salient during several interviews where participants had to talk about tough topics, thinking… 'wow I understand these responses'. Therefore, the quality of the data has been largely good so far, as I felt the authenticity in all,
*****

However, sometimes I reflect on being an early career researcher and wonder if my interview skills are good enough to elicit quality data. Nevertheless, according to the information power person [I later found this for my own information and to cite as Multerud et al., (2016)] interviewer skill is not the only thing that can determine quality of dialogue. These authors suggest that both the articulateness …and the "chemistry" between participant and interviewer also matter. I can say that without a doubt, both of these aspects have been strong so far. This is something that I need to talk about when addressing information power.

7/2025

I forgot to start recording the audio for the first few seconds of an interview!!!! Luckily, I was taking notes, but still it was not a good feeling to make this mistake. I'd say my confidence suffered and took some time to return during the interview…

Journal Reflexivity 4: July 2025

I did notice that in some interviews, participants would stray from the question being asked and it would derail the interview schedule for a moment. I would let them speak, but always wondered quietly how I could bring the conversation back to the question at hand.

*****

*****

t was challenging to keep these participants from going ahead of the question and then finding a way to re-ask questions that were either partially answered or not yet asked. I would often try to just restate or summarize their response, and ask them if this is what they were trying to say…

…[there were times] when I was so connected to their response that it triggered so many other questions – many of which were not necessarily tied to the research question, I would not let my curiosity take over, mostly in the interest of time. I could easily talk about this topic for hours. However, I was hyper conscious of participants' time and did not want to overstep.

Journal Reflexivity 5: Aug 2025

It's coming to the point where I need to decide if I should close recruitment. The idea of 'information power' is somewhat subjective, which I feel is both good and bad. Am I experienced enough to make this determination? Are my interview skills good enough to have elicited the depth needed across data? I think I am likely near the top end of information power, as many codes seem to be overlapping across participants now, and after 10 interviews it feels like I have a good number of variances in the codes as well. Conversations seemed to flow naturally, and almost all participants commented that they enjoyed the interview. *****

*****

However, I believe that the connections were genuine and my ability to build trust with participants, based on me revealing shared experiences… was vital in the positive outcomes of data collection. Some negative aspects included poor internet connection at times, and my perceived low self-efficacy in getting participants to stick to the question asked.

8/2025

A reflexive point to consider; specifically about participants' listing supervisory support (supervision) and inter-staff opportunities for shared experience support as consistent recommendations, is that maybe participants responded with these answers because we had been talking about things that were either supportive for them or not supportive for them in the previous question. And since [all] the interviews typically followed the interview schedule, this question generally came in the same place. Perhaps there is a sort of question ordering bias going on which influences the response to this question, and primes participants to list supervision or shared support? However, maybe it is also just a very legitimate and common issue that school staff face, since it appeared throughout the interviews at different points.

8/2025

*****

Journal Reflexivity 6: Sep 2025

I just had another realization while completing the analysis... Although much of the process was inductive, such that I relied on the actual words of participants as the primary source of the constructed meaning, my researcher lens, as the one looking for secondary trauma and reading about STS so deeply for the last several years, must have influenced the deductive aspect of analysis as well. Perhaps I am just applying STS theoretical models to the words of the participants? Is that the purpose of this type of research?

9/2025

I did some reading on RTA methodology, and it seems like it is fine to have some deductive analysis because it is based on STS theory. Ok. This makes sense. I feel a sense of relief moving forward.

Figure S2.

Interview Schedule/Guide

Introduction

Hello, my name is ***** and I am ***** school of psychology at ***** ***** Prior to this role, I worked within a school district in ***** as a ***** and I appreciate you speaking with me.

As covered in the information sheet you received, I would like to talk with you about your experiences of hearing about students'/pupils' potentially traumatic experiences.

The aim of this is to better understand the effects and experience of school personnels' exposure to this type of information, with the hopes of helping to better support individuals like you.

The interview should be no longer than 60 minutes. Once the interview begins, I will start audio recording. Do you agree to continue? Do you have any questions before we start?

Interview and recording begin.

Discussion/Question Guide: (reminder that privacy is important, please refrain from using actual names)

1. Can you tell me briefly about your job position and role in the school, and discuss what it involves (or involved if retired)?
   - Prompt: How long have you been in this role? What are some positives of this role? (PAUSE for response) any negatives?
   - Prompt if retired or left field: And why did you decide to leave?
2. Would you share with me your overall personal experiences of interacting with pupils during your job role, focusing on the types of interactions and your thoughts about them?
   - Prompt: Why do you believe that is? How often does this happen?
3. I would like to ask you about any specific instances of pupils/students telling you, or you knowing in another way about their adverse or potentially traumatic experience. Without stating actual names of people or locations, can you describe one or more times when this happened?
   - Prompt: How did you feel when this happened? How long did these feelings or emotions last?
4. If you had any, can you describe the effects (symptoms) that you believe were related to you hearing or learning about the student's adverse or potentially traumatic experiences?
   - Prompt: How did this affect your job function (work)?
   - Prompt: How did this affect your overall well-being (life)?

5. Can you share with me anything that you or anyone else did that you believe was helpful or supportive for you, after receiving this information from or about the pupil?

    - Prompt: Why do you think that this was helpful or supportive (how did you determine this)?
    - Prompt: Was there anything else that you think could have been helpful or supportive to you that was not done? OR was there anything that was unsupportive or unhelpful?

6. What is your opinion on the level of preparation (training) you had at the time of learning about the experience, and currently have for dealing with pupils' potentially traumatic experiences?

    - Prompt: Why do you think that is? In what ways did this contribute to how you responded or coped?
      Would you act or cope differently if this occurred again?

7. What advice would you give to other educators and education institution leaders to help them support themselves and their colleagues who hear about the trauma experiences of their students/pupils?

8. In closing, I would like to get your thoughts on some terms that are used in relation to professions like yours:
   When I say each term, can you please tell me anything that comes to mind or briefly describe any relations you have with them?
   If you are unfamiliar with any term or don't relate to it, feel free to say so:

    - Burnout
    - Empathy
    - Trauma-informed
    - Post-Traumatic Stress Disorder (PTSD)
    - Secondary Traumatic Stress (STS)
    - Resilience
    - Compassion Fatigue (CF)
    - Compassion Satisfaction (CS)

   Prompt: Can you give me an example?

   General prompts for entire interview:

    - "Can you tell me a bit more about that?"
    - "What was that like for you?"
    - "How did that make you feel?"
    - "What were you thinking during that time?"
    - "Could you give me an example of that?"
    - "What do you mean by…?"
    - "What did you think about that?"

Closing the interview

We have reached the end of my questions, thank you for taking part. Is there anything else you would like to add?

Are there any questions I have not asked, or information not shared that you think would be important for me to know?

- That brings us to the end of our interview, are you still happy for this data to be used in the study?

Ok, great. You can withdraw from this study up to two weeks from today. During the analysis process, your data will be made anonymous, and you will no longer be able to withdraw, as all identifiable information will be deleted.

If you decide you would like to withdraw, please contact ***** through our contact details which are included on the study information sheet.

- Thank you again for participating in this study, your involvement is very much appreciated. Would you like to be entered into a random drawing for a £50 ***** ***** gift voucher? If yes, please provide your email.

Figure S3.

Recruitment Materials

Study Invitation Letter

Dear School Staff,

Researchers from ***** would like to invite you to participate in a study on the experiences of school personnel who have heard about pupils' trauma experiences.

We are seeking to understand YOUR experiences of interacting with students/ pupils who may have told you (or you have come to know in another way) about their adverse or potentially traumatic experiences and learn how this impacted you.

We would like you to take part in a one-to-one online or in-person interview (your choice) to get your thoughts on this topic. Taking part in this study does not require that you miss any school periods or be on school property, as researchers will happily meet online, during your free time. The interview should last no longer than 60 minutes. No personal identifiable information will be reported in the study and a high standard of confidentiality will be practiced. This opportunity is voluntary, and you are <u>not</u> required to take part. However, your perspective could inform important future supports for school personnel. To show appreciation for voluntarily giving time, you will also be offered entry into a drawing for a chance to win a £50 ***** gift voucher, and a £5 donation made to the charity ***** for each participant (anonymously).

An information sheet about this research is attached to this email for your convenience. If you are willing to participate in this important research, please provide your email address and consent to be contacted via this form: ***** or scan the QR code below with your phone.

You may also emai ***** with any questions.

******

Thank you for reading this invitation.

Sincerely,

*****

Study information Sheet

School Personnels' Experiences of Secondary Trauma

Researchers from the School of Psychology ***** would like to invite you to take part in a study. Please read the information below carefully, as it details the purpose of this research and what your participation entails. *****

If you decide that you wish to participate, please provide your email via this form: ***** or scan this QR code so we may contact

For questions, email *****

What is the purpose of the Study?

Research has evidenced that school personnel may be impacted by hearing about the adverse or potentially traumatic experiences of their students/pupils. There is limited research coverage and understanding of how school personnel experience some of the effects of being exposed to this type of information. Yet, it is suggested in scientific literature that school personnel may experience the effects of this exposure differently than other professions (e.g. nurses, emergency service professions). Therefore, we would like to gain a deeper understanding of how school personnel experience and describe the effects of hearing about pupils' adverse or potentially traumatic experiences. As a current or past educator who learned about a pupil's potentially traumatic experience, your experiences, thoughts and feelings about this topic may be helpful in developing future supports for school personnel.

This research is being conducted ***** has been approved by the ***** ***** Research Ethics Committee

What will participation involve?

If you agree to participate, you will be invited to take part in a short, individual interview with the lead researcher. This can be either in-person or online video and will be scheduled at a time convenient for you. The interview should last no longer than 60 minutes. You will be asked some questions about your experiences of hearing about pupils' adverse experiences. Your responses will be transcribed; however, your participation is confidential and no identifiable information about you, or your place of work will be reported.

Am I required to participate?

No, your participation is voluntary, and you can freely choose not to take part, without any negative consequences. You may also withdraw from the study any time prior to and during your interview, even after signing the consent form. Due to the nature of interview data collection and anonymization for analysis, you are only able to withdraw up to two weeks AFTER your interview, as all names and identifiable information will be removed from transcripts. To withdraw, please contact a member of the research team, via the contact information included at the end of this document.

Are there any risks or benefits in my participation?

Your participation in this research will help us to increase our understanding of the experiences and effects that school personnel have after hearing about pupils' adverse experiences. While there may not be any immediate or direct benefit to you, it is hoped that this understanding will contribute to developing much needed supports for school personnel in the future. Furthermore, to show appreciation for your time, you will be offered entry into a drawing for a chance to win a £50 *****

gift voucher, and a £5 donation made to the charity *****
***** or each participant (anonymously).

There is a potential risk of you being upset or distressed during or after the interview, as discussing your experience may include sensitive topics. If you become distressed or upset, you will be allowed to take a break and either continue or terminate the discussion. You may be contacted prior to the scheduling of your interview to discuss this risk. We will also signpost supports if further care is needed.

What information will be collected about me and how will it be used?

If you decide to take part in the study, we will collect demographic information, such as your age, gender, job role, and years of experience. This information will be used only for the purposes of the research described in this document. During the interview, audio recording will occur for the purpose of transcription, after which the original audio will be destroyed. Transcription will be done with the aid of WhisperTranscribe, a secure artificial intelligence platform that does not retain audio after transcription (see WhisperTranscribe All identifiable information, such as specific names of individuals and locations, will be removed from the transcript. The information you provide will be confidential and not shared with anyone else, except in an instance where you share information indicating that you or another person is at risk of harm. In this case, appropriate emergency services will be contacted.

This research will be conducted in compliance with data protection legislation. For more information about how we look after your information, how to access your rights and who to contact if you have any queries or concerns about data protection, please visit the *****
*****

What will happen to the results of this study?

The pseudonymized results of this study will be used in part fulfilment of a PhD and will be submitted to scientific journals for publication. The results of the study may also be presented at conferences. All or part of the PhD which this study is towards might be made available to the public. However, participants' names and places of work are anonymized/ pseudonymized so individuals and schools will not be identifiable.

Who has reviewed this study?

This study has been reviewed by the Faculty of ***** Research Ethics Committee.

What if I have a complaint about the study?

If you have any concerns about any aspects of this research study, you can contact the Principal Investigator, Should you remain unhappy and wish to make a formal complaint, you can contact the Research Governance Team at *****
*****

Thank you for reading this information. Please see the research team's contact information below:

*****

*****

*****

Supplementary Materials (cont.)

Figure S4.

Data Analysis and Coding

The analytic process began with deep familiarization with the data, during which the first author repeatedly read transcripts, noting points that stood out and reflecting on their own interpretation of these points. After a significant period of reading and re-reading, and then stepping away from the transcripts, we began the systematic process of generating codes relevant to the research questions. Coding, also conducted by the primary author, involved copying relevant quotes from participants' transcripts whose sentiments captured the essential points they noted during data familiarization, then naming each code with a representative short title. Most codes embodied semantic elements of the data; however, some parts of coding, such as their names and descriptions, captured more latent features. This process was iterative and involved several weeks of revisiting transcripts, adding, deleting, naming, and renaming codes to produce a foundational dataset in the form of a coding table (Coding Table example below). From this foundation, we constructed themes by identifying and organizing patterns of shared meaning across the coding table. Additionally, themes were constructed from codes that differed in co-constructed meaning, such as sentiments that were distinct or opposed to most others. This process also involved a recursive approach to developing, reviewing, and refining the themes, ensuring they told a coherent story grounded in the participants' own words. The Theme Map is illustrated in Figure S5; Thematic Map).

Coding Table Example

RQ	THEME	CODES	Illustrative Excerpts (Verbatim)
(a) What are the experiences of STS in school personnel? (b) How do school personnel deal with any effects... and what are the sources of support?	**A System Under Strain: Institutional Failures and Inadequate Support**	Academics vs. Socioemotional Wellbeing Conflict	…it should not be 90 [%] academic and 10% how to be a person, you know, it needs to be right, closer to 50/50. And... that brings me into conflict with other colleagues and the principal...- William | ...it's just more the external factors that really do make it negative... the paperwork, the, the pressure to get them to write their name or to, you know, to count to 10...-Maggie
		Critique of Performative Institutional Language	It's individual centered. It's not systemic. It's an individual burns out. The system doesn't burn you out. It really makes me angry… [Resilience is] a really awful HR word. Masculine word...-Claire | People love words. It's the same with, like... diversity and inclusion. But then, you know, in practice that's maybe not as, as easy and as nice as it sounds…-Kelly
		Feeling unheard/unsupported by leadership	…there was an inflexibility to listen from management. I think the pressures from Ofsted and performing were such that they just felt like, you just got to get it done.-Lee | I'm sure someone from management would argue with me, but that's my experience.-Kelly
		Lack of Trauma-Informed Support FOR STAFF	But again, trauma affects everyone. Trauma doesn't just affect children... there is nothing in place to sort of support people in dealing with kind of hearing about trauma, I think.-Kelly | We spend all this time thinking about how to care for the kids... And we will have meeting after meeting... very little consideration goes into how to care for the adults.-Claire
		Perceived Inauthenticity of Formal Support	…we have access to counselling and stuff like that if we need it, but I do feel like that's just to tick the box... Personally myself, if I was going through something, I'd be more likely to go to my own GP...-Bonnie | I think the hotlines and all of that stuff is a waste of my time. It's a waste of my time... They don't know me. They don't know my students. They don't get it.-Ashley
		Shame-Based Culture Discouraging Help-Seeking	…the culture is still kind of shame based. So if you're not doing well in your behaviour management, then whoa, you know there's something wrong with you... I don't know if I want to sit too close to you... because I might catch whatever you've got…-Lee | I don't feel in a way like I can show when I'm stressed... you're meant to be, like, the calming influence in the room... you can't really show that you're stressed or struggling.-Dana
		Systemic Time Pressure & Role Overload	...there isn't actually time in our jobs for us to take that time to, you know, like decompress after you've had to deal with something.-Barbara | Oh, gosh, absolutely not. No, no, definitely not. [Is one day a week enough for principal duties?] You're pulled from pillar to post.-Bonnie
		Under preparedness for a difficult role	I know how to teach long multiplication... but that whole social and emotional piece is somewhat airy fairy… I'd say we had no preparation for working with children who were coming from difficult situations at home.-Bonnie | I think in college you do a bit of child protection training and that's it... it's just very brief. I don't feel like I was really prepared.-Dana
(a) What are the experiences of STS in school personnel?	**The Intrusive Weight of Trauma: An Emotional and Personal Toll**	Complex Emotional Cocktail: Disgust, Guilt, Anger, Sadness, Worry, and Hindsight	Very disgusted. I was like, that's just, that's awful...but it also makes sense. But I'm like, I feel bad that I hadn't, you know, known about it previous.-Dana | It's honestly hand in my heart when I hear that a child is being...neglected...I feel really angry... You would go home and you would worry.-Maggie
		Intrusive Traumatic Imagery	..those images gone through my head that the... mom had so graphically described were deeply disturbing and I couldn't, I could not shake those images out of my head. It was like I was watching hammer horror films in my head...-William | ...although I, I replay it in my mind, [pause] I send a lot of love to my inner child. Like a lot of love.-Ashley
		Irritability towards perceived non-traumatized students	I felt at the time... almost like cross with the other kids if they were rude or did something wrong, because it's like you've got everything great for you...-Kelly | ...sometimes I am less empathetic to students...who are just being brats...and I'm a bit less patient and I can feel that in my body. And that's not me.-Ashley
		Lasting Guilt and Responsibility for Former Students	I have often cried over that boy because I think, like, oh, if I hadn't, like, I left... I wasn't going to be able to change the direction of his life. But yeah, I often think of him…-Kelly | ...looking into my child's eyes and, and thinking why can't other children have what you have?-William
		Mirrored Trauma: Staff's Past in Student's Present	I often see this kind of mirror, mirrored thing happening here... I did say to her, I know what that feels like... So I see you, and I'm sorry that you have to go through that.-Ashley | ...it's being the one to be calm. That part was triggering for me...I've done that as a child...it just, it was like, damn, I'm really, I'm this person.-Ashley
		Physical Manifestation: Reliving Past Trauma	Yeah, for myself, I had a really strong, like, emotional, Physical, emotional response that I remembered from when my own father had a heart attack... It was a very similar feeling. I recognized the feeling, actually.-Claire | ...it can still have quite a physical effect on me, like remembering that that horrible event... there's definitely still like almost a physical kind of response to it rather than just thinking about it.-Kelly

Note: Fig. S4 describes the process we used during coding, and how the subsequent themes in Fig. S5 below were developed from a set of generated convergent codes.

[To maximize readability of Figure S5. we placed the map on its own page below**]**

Figure S5.

Thematic Map

Note: Fig. S5 maps the complete process of constructing themes from the generated codes and their connection to the research question

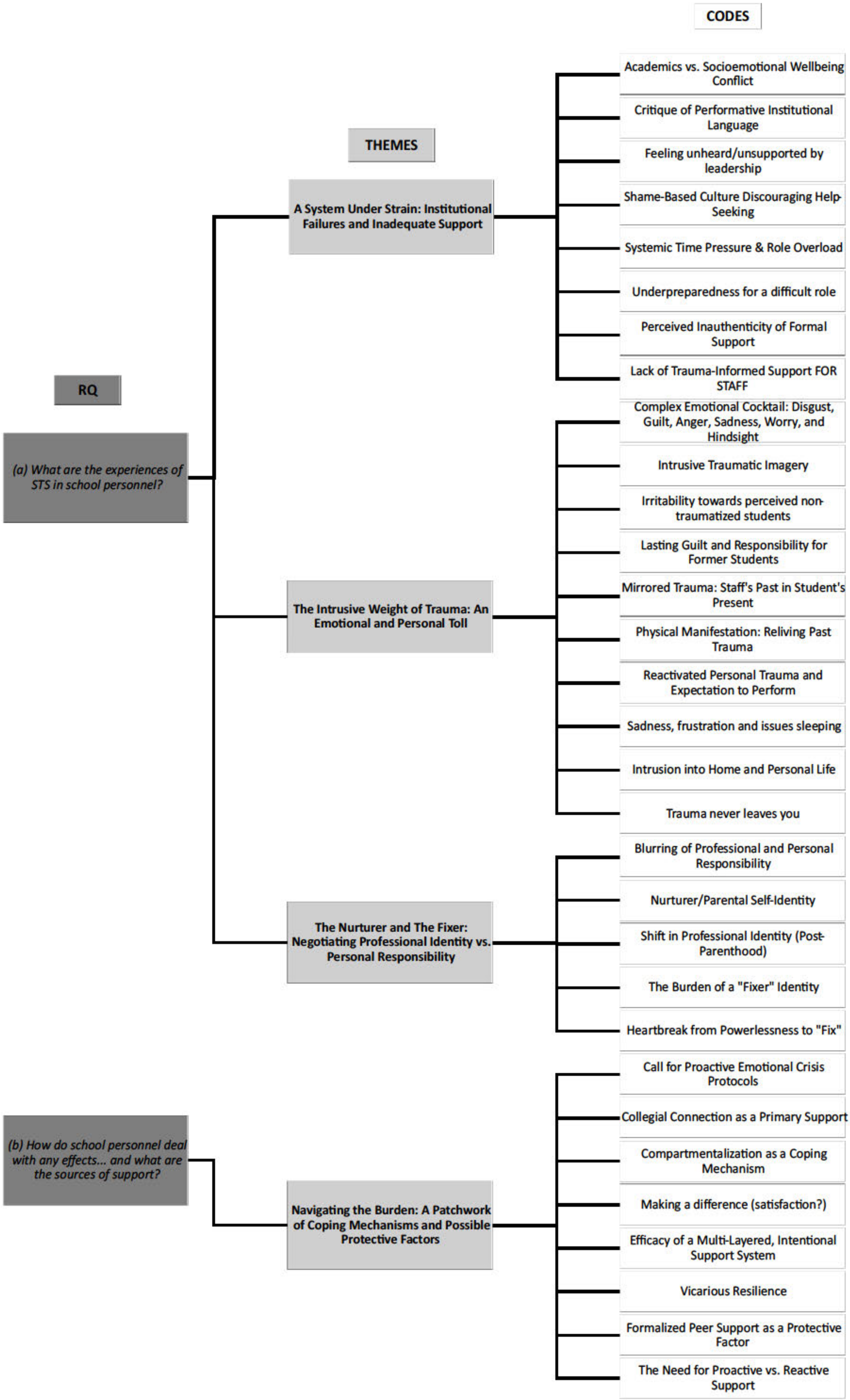
CODES
THEMES
RQ
(a) What are the experiences of STS in school personnel?
A System Under Strain: Institutional Failures and Inadequate Support
Academics vs. Socioemotional Wellbeing Conflict
Critique of Performative Institutional Language
Feeling unheard/unsupported by leadership
Shame-Based Culture Discouraging Help-Seeking
Systemic Time Pressure & Role Overload
Underpreparedness for a difficult role
Perceived Inauthenticity of Formal Support
Lack of Trauma-Informed Support FOR STAFF
The Intrusive Weight of Trauma: An Emotional and Personal Toll
Complex Emotional Cocktail: Disgust, Guilt, Anger, Sadness, Worry, and Hindsight
Intrusive Traumatic Imagery
Irritability towards perceived non-traumatized students
Lasting Guilt and Responsibility for Former Students
Mirrored Trauma: Staff's Past in Student's Present
Physical Manifestation: Reliving Past Trauma
Reactivated Personal Trauma and Expectation to Perform
Sadness, frustration and issues sleeping
Intrusion into Home and Personal Life
Trauma never leaves you
The Nurturer and The Fixer: Negotiating Professional Identity vs. Personal Responsibility
Blurring of Professional and Personal Responsibility
Nurturer/Parental Self-Identity
Shift in Professional Identity (Post-Parenthood)
The Burden of a "Fixer" Identity
Heartbreak from Powerlessness to "Fix"
(b) How do school personnel deal with any effects... and what are the sources of support?
Navigating the Burden: A Patchwork of Coping Mechanisms and Possible Protective Factors
Call for Proactive Emotional Crisis Protocols
Collegial Connection as a Primary Support
Compartmentalization as a Coping Mechanism
Making a difference (satisfaction?)
Efficacy of a Multi-Layered, Intentional Support System
Vicarious Resilience
Formalized Peer Support as a Protective Factor
The Need for Proactive vs. Reactive Support
