# Supplementary material for: “You Are Left Holding Their Despair”: A Reflexive Thematic Analysis of Secondary Trauma Among School Staff
Source: J Child Adolesc Trauma. 2026 Sep 9;19(4):2669–85. doi: 10.1007/s40653-026-00972-6 (PMC13737240; doi:10.1007/s40653-026-00972-6)
Supplement: Supplementary file 2 [file 40653_2026_972_MOESM2_ESM.pdf]

Researcher Positionality and Reflexivity

In line with qualitative methods and the principles of RTA, this study was underpinned by a continuous reflexive process (Trainor & Bundon, 2021). The use of first-person pronouns reflects the primary author's positionality, aligning with Braun and Clarke's (2024) advocacy for methodologically coherent first-person writing in RTA. Guided by Finlay's (2002) conceptualization of reflexivity, the approach blended personal introspection, intersubjective reflection on the researcher-participant dynamic, and a social critique of the research context and process itself. This allowed us to leverage the primary author's personal experiences as a source of latent insight while co-constructing meaning and remaining conscious of power imbalances (Finlay, 2002). Fieldwork notes and reflexive journal excerpts provide an audit trail in the online supplementary materials (Figure S1, Reflexive Journal and Notes).

My positionality as a former school staff member in a student support role was central, serving as both a foundation for investigation and a lens for analysis. This insider status required conscious introspection to navigate the closeness. Embracing past experiences facilitated a deeper understanding of the participants' worlds; as noted in my journal, "…the sighs, the deep breaths between the [things]…said" are also meaningful. However, this closeness presented personal challenges, occasionally prompting me to step away from transcripts to gain perspective. Another layer of reflexive consideration surrounds my position as a former U.S.-based practitioner working with participants based in Ireland, Northern Ireland, and England. This could have introduced a degree of cultural distance that was beneficial for analysis, potentially reducing the risk of taking certain contextual and cultural differences for granted. However, this outsider-within position could also have introduced blind spots, such as assumptions shaped by my exposure to state- and district-managed school structures in the U.S., and a distance from culturally significant events like the legacy of the Troubles in Northern Ireland. While such topics were present in interviews, these blind spots could have influenced the questions asked (and not asked) and the co-constructed meaning of responses. These are limitations I attempted to hold consciously throughout the analytic process. Concrete reflexive strategies employed included sustained reflexive journaling, periodic deliberate

distancing from transcripts, and iterative check-ins with the research team at key stages of the analysis.

Intersubjective reflection focused on the co-construction of knowledge and the participant "chemistry" vital for data quality. This occasionally created tension between fostering open dialogue and adhering to time constraints. Reflexivity also extended to practical challenges. Initial recruitment proved a "frustrating lesson," necessitating a pragmatic shift from random to convenience and snowball sampling. This critical reflective lens was maintained throughout the analysis.

The positionality of the contributing authors also warrants acknowledgment. The second author is a professor of health psychology with extensive qualitative and quantitative research experience; the third author is an educational psychologist with substantial research experience in school-based contexts; and the fourth author is a professor of mental health psychology with a sustained research focus on psychological trauma. Their collective disciplinary backgrounds in health, education, and trauma psychology, along with their contextual and cultural knowledge, provided perspectives different from my own, and this difference was a resource throughout the study. Through iterative reviews of drafts, in-person supervisory meetings, and ongoing collaborative dialogue, their input shaped the study's conceptual framing, the writing, and the balance of interpretations. While their positionality is best understood through the lens of their professional and disciplinary expertise rather than through direct proximity to the data, their engagement with developing the manuscript may be understood as a form of analytical scaffolding that helped me to test, refine, and situate the interpretive work carried out in the primary analysis.

References

Braun, V., & Clarke, V. (2024). Supporting best practice in reflexive thematic analysis reporting in *palliative medicine*: A review of published research and introduction to the *reflexive thematic analysis reporting guidelines* (RTAG). *Palliative Medicine*, *38*(6), 608–616. https://doi.org/10.1177/02692163241234800

Finlay, L. (2002). Negotiating the swamp: The opportunity and challenge of reflexivity in research practice. *Qualitative Research*, *2*(2), 209–230. https://doi.org/10.1177/146879410200200205

Trainor, L. R., & Bundon, A. (2021). Developing the craft: Reflexive accounts of doing reflexive thematic analysis. *Qualitative Research in Sport, Exercise and Health*, *13*(5), 705–726. https://doi.org/10.1080/2159676X.2020.1840423
